# Utility of icteric index in clinical laboratories: more than a preanalytical indicator

**DOI:** 10.11613/BM.2021.020703

**Published:** 2021-04-15

**Authors:** Rufino Mondejar, María Mayor Reyes, Enrique Melguizo Madrid, Consuelo Cañavate Solano, Santiago Pérez Ramos

**Affiliations:** 1 UGC Laboratory, Puerto Real University Hospital, Cádiz, Spain; 2 Biomedical Research Networking Centers in Cancer (CIBERONC), Madrid, Spain; 3 Committee of Personalised Medicine, Spanish Association of Medical Biopathology - Laboratory Medicine (AEBM-ML), Madrid, Spain

**Keywords:** jaundice, bilirubin, hyperbilirubinemia, haemolysis, receiver operating characteristic curve

## Abstract

**Introduction:**

Total bilirubin tests are highly demanded in clinical laboratories. Since icteric index (I-index) has zero cost, we aimed to evaluate its clinical utility and cost-effectiveness to determine if total bilirubin is necessary to be tested. We took into account if haemolysis could interfere to icteric index determination.

**Material and methods:**

Retrospectively we reviewed I-index results in two cohorts (43,372 and 8507 non-haemolysed and haemolysed samples, respectively). All determinations were done using Alinity c chemistry analysers (Abbott Diagnostics). Receiver operating characteristic (ROC) curve was used to determine the optimal index cut-off to discriminate between normal and abnormal bilirubin concentration (20.5 µmol/L).

**Results:**

The ROC curve analysis suggested 21.4 µmol/L as the optimal I-index cut-off but differences in sensitivity and specificity were detected between patient derivation. For rejecting purpose, 15.4 µmol/L and 17.1 µmol/L I-index thresholds were selected based on patient derivation (inpatients and emergency room; and primary care and outpatients, respectively) with 97% sensitivity and 0.25% false negative results. Sensitivity was much lower in haemolysed samples. We selected 34.2 µmol/L I-index as threshold to detect hyperbilirubinemia with 99.7% specificity and 0.26% false positive results, independent of haemolysis. With the icteric index cut-offs proposed, we would save 66% of total bilirubin requested and analyse total bilirubin in around 2% of samples without total bilirubin requested.

**Conclusions:**

This study supports the use of I-index to avoid bilirubin determination and to identify patients with hyperbilirubinemia. This work considers that the economic and test savings could help to increase the efficiency in clinical laboratories.

## Introduction

Along last decade, haemolysis, icterus, lipaemia (HIL) serum indices have been implemented in clinical laboratories, according to CLSI C56A guideline (*1*). Clinical chemistry analysers measure HIL indices under different wavelengths, providing approximate concentrations of haemoglobin, bilirubin and lipids in samples. These measurements are cheap, fast and objective and, more important, reduce the possibility of errors in the preanalytical phase which increases the patient safety (*2*-*4*).

Depending on manufacturers, measurement of serum indices requires a specific reagent or only saline solution or water. HIL index is not directly reported on laboratory reports since it does not guide a clinical action and are commonly used to decide if the result of a certain laboratory parameter is rejected, reported or reported with an annotation (*5*, *6*). There is a striking debate regarding the most appropriate and reliable strategy for dealing with pre-analytically altered laboratory test results, especially of those obtained in haemolysed samples (*7*-*10*). Besides this, there is heterogeneity in procedures on how to act upon haemolytic, icteric and lipaemic samples (*11*). Nonetheless, few studies have focused over other uses of icteric index (I-index).

Hyperbilirubinemia is defined in adults as having higher values than 20.5 µmol/L of total serum bilirubin. Hyperbilirubinemia can be isolated or associated to hepatitis, cirrhosis, haemolytic disorders, several inherited enzyme deficiencies, autoimmune liver diseases and conditions causing hepatobiliary obstruction (*12*). Total bilirubin (TBil) is one of the most requested parameters in a clinical laboratory (*13*). Despite TBil being inexpensive, the fact is that I-index is cheaper or has a “zero cost” make us to think if TBil is necessary to be tested.

For this reason, this study aim to evaluate the clinical utility and cost-effectiveness of I-index as, firstly, screening of TBil in order to reject the determination of this parameter and, secondary, detection of hyperbilirubinemia in those cases without TBil ordered. We also address the question if haemolysis can interfere in I-index determination.

## Material and methods

### Study design

This retrospective cross-sectional study was conducted at the Central Laboratory of the Puerto Real University Hospital in accordance with the Declaration of Helsinki and was approved by the ethical committee of the Biomedical Research of Andalusia, Spain (CCEIBA). Two cohorts were used in this study representing non-haemolysed (2-months period, September to October 2019) and haemolysed samples (5-months period, September 2019 to February 2020). We selected a larger period in haemolysed samples due to the relative low number of samples compared to non-haemolysed samples. We followed Standards for Reporting of Diagnostic Accuracy Studies (STARD) guideline to ensure that all relevant information is presented in the study (*14*).

### Subjects

In the non-haemolysed cohort, we extracted data of 43,372 samples from Laboratory Information System (LIS) MODULAB (version 3.1.02C, Werfen Group, Barcelona, Spain). Samples with haemolysis index (H-index) > 0.6 g/L were defined as haemolysed (Personnel communication, Abbott Laboratories, IL, USA) and were excluded from this cohort. We categorized four subgroups according to patient derivation (primary care (N = 23,720), outpatients (N = 9914), hospital inpatients [inpatients] (N = 4420) and patients who went to emergency room [emergency room] (N = 5318)). On the other hand, in the haemolysed cohort we extracted data of 8507 samples from LIS excluding samples with H-index ≤ 0.6 g/L (primary care (N = 3347), outpatients (N = 1754), inpatients (N = 1025) and emergency room (N = 2381)). Neonatal samples were excluded in both cohorts due to having a higher reference interval of bilirubin than adults. No other exclusion criterion was taken into account. Lipaemia was not studied in this work due to the very low number of lipaemic samples (0.13% of samples in 2-month period, data not shown). Laboratory parameter values, such as serum indices and TBil were obtained from the LIS.

### Methods

All venous blood samples were collected in standard plasma or serum tubes (Vacuette Tube Cat 9 mL Cat Serum Separator Clot Activator or Vacuette Tube 4 mL LH Lithium Heparin; Greiner Bio-One GbmH, Kremsmünster, Austria) and centrifuged at 3500xg for 7 min at 18 °C. Samples were processed in Alinity c chemistry analysers (Abbott Laboratories, Illinois, USA). Total bilirubin concentration was determined using Total Bilirubin Reagent Kit (ref. 04V51, Abbott Laboratories, Illinois, USA) and results was expressed in μmol/L. Reagent was calibrated using Alinity c Bilirubin Cal (ref. 8P61-01, Abbott Laboratories, Illinois, USA) each 336 hours or after reagent lot change and internally controlled daily using Technopath Multichem S Plus (ref. 08P88-10/-11/-12). HIL index was measured with a commercial saline solution (NaCl 0.9%, 500 mL, ref. 616003.9, Fresenius-Kabi, Barcelona, Spain) and its quantitative results were given in g/L of haemoglobin (H-index), µmol/L of bilirubin (I-index) or of added intralipid (L-index) (SI units), according to manufacturer´s instructions. Alinity c utilizes the same wavelengths as Architect c8000/c16000 (H-index: 500/524 nm; I-index: 572/604 nm and 628/660 nm; L-index: 524/804 nm) (*15*). Total bilirubin concentration and HIL indices were externally controlled once time per month using the Biochemistry Serum and Serum Indices Programmes, respectively, provided by The External Quality Control Programme from the Spanish Society of Laboratory Medicine (SEQC-ML). Data were collected from LIS by the MODULAB Exportation Module, which generated an Excel file.

### Statistical analysis

Statistical analysis was performed using the statistical software SPSS for Windows (v15) (IBM SPSS, NY, USA) and R software (3.6.3). We compared I-index and TBil test results. Total bilirubin concentration was considered as the gold standard. Deming regression was run in R software using ‘Rcmdr’ and ´deming´ package to perform regression between TBil and I-index. Bland-Altman was used to analyse the agreement between variables and was plotted in SPSS. Receiver operating characteristic (ROC) curve analysis was used to determine the optimal icteric index cut-off value to discriminate between patients with normal and abnormal bilirubin values. Total bilirubin concentration were considered abnormal when they were above 20.5 µmol/L. We calculated sensitivity, specificity, positive predictive value (PPV), negative predictive value (NPV), likelihood ratios for positive (LR+), negative (LR-), false positive (FP) and false negative (FN) results. All parameters were calculated for the whole population and for the four subgroups according to patient derivation. ANOVA test was used for mean comparison.

## Results

Data from the non-haemolysed cohort included 43,372 samples with I-index results. After initial analysis, neonatal samples and samples without TBil requested were excluded, remaining 22,485 samples (51.8%). Icteric index was well correlated to TBil (Supplementary Figure 1). The ROC curve analysis showed that the optimal I-index cut-off value to discriminate abnormal bilirubin was 21.4 µmol/L (Supplementary Figure 1). The sensitivity, specificity, PPV, NPV, LR+, LR- of I-index ≤ 21.4 µmol/L showed differences between primary care and outpatients, and inpatients and emergency room (Table 1). Then, we compared TBil values between the four types of patients and detected differences statistically significant between them (Supplementary Figure 2). In order to set the best cut-off to avoid TBil test (the highest sensitivity, the lowest FN rate and the highest number of tests saved), we analysed different cut-offs related to the differences between type of patients (data not shown). We selected two different cut-offs, an I-index cut-off ≤ 17.1 µmol/L for primary care and outpatients and ≤ 15.4 µmol/L for emergency room and inpatients. With these I-index cut-offs adjusted per type of patient, we obtained 97.0% sensitivity, NPV 99.6% and 0.28% of FN rate (Table 2). We analysed false negative results obtaining a range of TBil values of 22.2-29.1 µmol/L in most cases but three samples of a same impatient showed TBil results higher than 34.2 µmol/L (data not shown). So, to our first objective of this study, the use of these cut-offs would save 66% of TBil determinations.

**Table 1:** Sensitivity, specificity, positive and negative predictive values, positive and negative likelihood ratios, false negative and false positive results, for an I-index ≤ 21.4 µmol/L in different subgroups of patients

	**Primary care**	**Outpatients**	**Inpatients**	**Emergency room**	**All samples**
Sensitivity, % (95% CI)	96.5 (95.1 to 97.8)	91.6 (88.6 to 93.9)	76.3 (72.7 to 79.5)	76.5 (71.9 to 80.6)	86.3 (84.8 to 87.7)
Specificity, % (95% CI)	84.1 (83.4 to 84.8)	87.9 (87.0 to 88.8)	96.8 (96.1 to 97.4)	94.6 (93.6 to 95.4)	87.9 (87.4 to 88.3)
PPV, % (95% CI)	30.15 (28.3 to 32.6)	38.64 (35.9 to 41.7)	85.47 (82.2 to 88.2)	67.4 (62.7 to 71.8)	42.6 (41.1 to 44.1)
NPV, % (95% CI)	99.7 (99.6 to 99.8)	99.2 (98.9 to 99.4)	94.3 (93.4 to 95.2)	96.5 (95.7 to 97.2)	98.4 (98.2 to 98.6)
LR+ (95% CI)	6.07 (5.79 to 6.36)	7.58 (7.00 to 8.20)	23.98 (19.19 to 29.97)	14.12 (11.86 to 16.81)	7.12 (6.837 to 7.416)
LR- (95% CI)	0.04 (0.03 to 0.06)	0.10 (0.07 to 0.13)	0.25 (0.21 to 0.28)	0.25 (0.21 to 0.30)	0.16 (0.14 to 0.17)
False negatives, N (%)*	26 (0.24)	36 (0.65)	143 (4.68)	85 (3.00)	290 (1.29)
False positives, N (%)*	1636 (14.83)	621 (11.16)	78 (2.55)	134 (4.73)	2469 (10.98)
Samples, N (%)^†^	11,028 (49.06)	5567 (24.77)	3049 (13.56)	2835 (12.61)	22,479 (100)
*Percentage relative to its subgroup. ^†^Percentage relative to all samples studied in this cohort with Tbil requested. PPV - positive predictive values. NPV - negative predictive value. LR+ - positive likelihood ratio. LR- - negative likelihood ratio. CI – confidence interval. I-index – icteric index. Tbil – total bilirubin.					

**Table 2:** Sensitivity, specificity, positive and negative predictive values, positive and negative likelihood ratios, false negative and false positive results, adjusted by I-index cut-off in different subgroups of patients

	**Primary care**	**Outpatients**	**Inpatients**	**Emergency room**	**All samples**
	**I-index ≤ 17.1 µmol/L**		**I-index ≤ 15.4 µmol/L**		
Sensitivity, % (95% CI)	99.6 (98.8 to 99.9)	97.4 (95.5 to 98.6)	92.7 (90.3 to 94.5)	98.6 (96.8 to 99.4)	97.0 (96.2 to 97.7)
Specificity, % (95% CI)	67.3 (66.4 to 68.2)	74.5 (73.3 to 75.7)	86.8 (85.4 to 88.1)	79.1 (77.4 to 80.6)	72.9 (72.3 to 73.5)
PPV, % (95% CI)	17.8 (16.6 to 19.0)	24.1 (22.1 to 26.2)	63.3 (60.1 to 66.5)	40.8 (37.6 to 44.1)	27.2 (26.2 to 28.2)
NPV, % (95% CI)	100.0 (99.9 to 100.0)	99.7 (99.5 to 99.8)	98.0 (97.3 to 98.5)	99.7 (99.4 to 99.9)	99.6 (99.5 to 99.7)
LR+ (95% CI)	3.04 (2.96 to 3.13)	3.82 (3.63 to 4.01)	7.04 (6.34 to 7.81)	4.71 (4.36 to 5.09)	3.58 (3.49 to 3.66)
LR- (95% CI)	0.01 (0.00 to 0.02)	0.04 (0.02 to 0.06)	0.08 (0.06 to 0.11)	0.02 (0.01 to 0.04)	0.04 (0.03 to 0.05)
False negatives, N (%)*	3 (0.03)	11 (0.20)	44 (1.44)	5 (0.18)	63 (0.28)
False positives, N (%)*	3370 (30.56)	1312 (23.57)	323 (10.57)	518 (18.27)	5523 (24.57)
Samples, N (%)^†^	11,028 (49.06)	5567 (24.77)	3049 (13.56)	2835 (12.61)	22,479 (100.00)
Test saved, N (%)^†^	6926 (30.81)	3828 (17.03)	2130 (9.48)	1955 (8.70)	14,839 (66.01)
*Percentage relative to its subgroup. ^†^Percentage relative to all samples studied in this cohort with Tbil requested. PPV - positive predictive values. NPV - negative predictive value. LR+ - positive likelihood ratio. LR- - negative likelihood ratio. CI – confidence interval. I-index – icteric index. Tbil – total bilirubin.					

To address the second objective of this study we analysed again the non-haemolysed cohort selecting samples with I-index and TBil results using an I-index cut-off ≥ 34.2 µmol/L. We obtained 99.7% specificity and 0.26% FP results (Table 3). Then, we focused our study in samples with I-index without TBil requested (N = 20,887, 48.2%; non-haemolysed cohort). Out of them, 447 samples (2.1%) had I-index ≥ 34.2 µmol/L (Table 4).

**Table 3:** Sensitivity, specificity, positive and negative predictive values, positive and negative likelihood ratios, false negative and false positive results, for I-index cut-offs ≥ 34.2 µmol/L in different subgroups of patients

	**Primary care**	**Outpatients**	**Inpatients**	**Emergency room**	**All samples**
Sensitivity, % (95% CI)	28.4 (25.3 to 31.8)	25.1 (21.2 to 29.4)	42.0 (38.2 to 46.0)	23.8 (19.7 to 28.4)	30.8 (28.9 to 32.8)
Specificity, % (95% CI)	99.6 (99.5 to 99.7)	99.7 (99.5 to 99.8)	99.9 (99.6 to 100.0)	99.9 (99.7 to 100.0)	99.7 (99.6 to 99.8)
PPV, % (95% CI)	83.9 (78.8 to 87.9)	88.4 (81.5 to 93.0)	98.8 (96.6 to 99.6)	97.7 (92.1 to 99.4)	91.7 (89.5 to 93.5)
NPV, % (95% CI)	95.1 (94.7 to 95.5)	94.1 (93.5 to 94.7)	87.5 (86.3 to 88.7)	90.0 (88.8 to 91.0)	93.3 (92.9 to 93.6)
LR+ (95% CI)	73.14 (52.58 to 101.73)	92.00 (53.17 to 159.18)	343.64 (110.48 to 1068.88)	293.75 (72.61 to 1188.35)	106.32 (81.76 to 138.25)
LR- (95% CI)	0.72 (0.69 to 0.759)	0.75 (0.71 to 0.79)	0.58 (0.54 to 0.62)	0.76 (0.72 to 0.81)	0.69 (0.68 to 0.71)
False negatives, N (%)*	524 (4.75)	320 (5.75)	349 (11.42)	276 (9.74)	1469 (6.53)
False positives, N (%)*	40 (0.36)	14 (0.25)	3 (0.10)	2 (0.07)	59 (0.26)
Samples, N (%)^†^	11,028 (49.05)	5567 (24.76)	3055 (13.59)	2835 (12.61)	22,485 (100.00)
*Percentage relative to its subgroup. ^†^Percentage relative to all samples studied in this cohort with Tbil requested. PPV - positive predictive values. NPV - negative predictive value. LR+ - positive likelihood ratio. LR- - negative likelihood ratio. CI – confidence interval. I-index – icteric index. Tbil – total bilirubin.					

**Table 4:** Samples with I-index without TBil requested in 2-months period

	**Primary care**	**Outpatients**	**Inpatients**	**Emergency room**	**All samples**
Samples, N (%)*	12,692 (60.77)	4347 (20.81)	1365 (6.54)	2483 (11.89)	20,887 (100)
Samples I-index ≥ 34.2 µmol/L, N (%)^†^	291 (2.29)	85 (1.96)	37 (2.71)	34 (1.37)	447 (2.14)
*Percentage relative to all samples studied in this cohort without Tbil requested. ^†^Percentage relative to its subgroup. I-index – icteric index. Tbil – total bilirubin.					

In order to evaluate whether haemolysis could interfere the use of I-index, we used data from the haemolysed samples cohort. Total bilirubin was requested in 4630 samples (54.4%). Using the above proposed rejection cut-offs we observed a strong decrease in sensitivity (79.2%) and higher false negative rate (2.1%) (Table 5). On the other hand, we selected the I-index cut-off for screening of hyperbilirubinemia (≥ 34.2 µmol/L) and found similar results than in non-haemolysed samples (specificity > 99.5% and 0.4% FP results, data not shown).

**Table 5:** Sensitivity, specificity, positive and negative predictive values, positive and negative likelihood ratios, false negative and false positive results, adjusted by I-index cut-off in different subgroups of patients in haemolysed samples (H > 0.6 g/L)

	**Primary care**	**Outpatients**	**Inpatients**	**Emergency room**	**All samples**
	**I-index ≤ 17.1 µmol/L**		**I-index ≤ 15.4 µmol/L**		
Sensitivity, % (95% CI)	81.3 (71.1 to 88.5)	72.4 (59.8 to 82.3)	82.9 (75.3 to 88.6)	78.0 (71.8 to 83.2)	79.2 (75.2 to 82.6)
Specificity, % (95% CI)	87.3 (85.5 to 88.9)	89.6 (87.5 to 91.4)	93.6 (91.1 to 95.4)	94.1 (92.6 to 95.3)	90.5 (89.6 to 91.4)
PPV, % (95% CI)	24.0 (19.2 to 29.6)	30.0 (23.0 to 38.0)	75.0 (67.1 to 81.5)	69.0 (62.2 to 74.7)	47.8 (44.2 to 51.3)
NPV, % (95% CI)	99.0 (98.3 to 99.4)	98.1 (97.0 to 98.9)	95.9 (93.9 to 97.3)	96.2 (95.0 to 97.2)	97.6 (97.0 to 98.0)
LR+ (95% CI)	6.39 (5.39 to 7.58)	6.96 (5.44 to 8.90)	12.88 (9.21 to 18.00)	13.24 (10.43 to 16.81)	8.37 (7.53 to 9.29)
LR- (95% CI)	0.21 (0.13 to 0.34)	0.31 (0.20 to 0.47)	0.18 (0.12 to 0.27)	0.23 (0.18 to 0.30)	0.23 (0.19 to 0.28)
False negatives, N (%)*	14 (0.88)	16 (1.60)	21 (3.23)	44 (3.17)	95 (2.05)
False positives, N (%)*	193 (12.13)	98 (9.80)	34 (5.22)	70 (5.04)	395 (8.53)
Samples, N (%)^†^	1591 (34.36)	1000 (21.60)	651 (14.06)	1388 (29.98)	4630 (100)
*Percentage relative to its subgroup. ^†^Percentage relative to all samples studied in this cohort with Tbil requested. PPV - positive predictive values. NPV - negative predictive value. LR+ - positive likelihood ratio. LR- - negative likelihood ratio. CI – confidence interval. I-index – icteric index. Tbil – total bilirubin. H – haemolysis index.					

## Discussion

To our knowledge, this is the first study that evaluates I-index on Alinity c. It is well known that the icteric index is correlated to total bilirubin but we checked it in our platform (*16*-*19*). Several studies have been published with the objective of reducing number of TBil tests with two main platforms, Roche and Abbott (*16*-*21*). An interesting study of HIL index in these two platforms showed an unacceptable comparability between them (*15*). In terms of sensitivity, FN rate and NPV, Abbott platform seems to be slightly less efficient than Roche to save tests (Table 6). Pasqualetti *et al*. reported higher sensitivity (99.0%) and similar FN results (≤ 0.2%) with lower I-index (13.7 µmol/L) on an Abbott platform (*19*). It would be reasonable to think that if we lowered the cut-off we would obtain better sensitivity data and fewer FN results, but we would significantly reduce the number of TBil saved, as it is shown by Pasqualetti and co-workers (about 35-40% of TBil saved) (*19*). With our proposed I-index cut-offs we would save 66% of all TBil tests requested when it is not clinically relevant and saved 1023 € in two months (0.069 € per test). We found differences in sensitivity and FN results at the same cut-off between inpatients and outpatients. A reason why we can find these differences was the utilization of serum and plasma samples (*17*). In our laboratory, serum samples are usually used in primary care and outpatients while plasma samples in emergency room and inpatients. Nonetheless, we could not separate the type of tube to perform a further analysis.

**Table 6:** Comparative analysis across different platforms choosing the best I-index cut-off with intention to save the highest proportion of samples with TBil ordered with the less false negative results

**Study (source)**	**Platform**	**I-index cut-off**	**Sensitivity**	**FN**	**NPV**	**N**	**Test saved**
Arbiol-Roca *et al.* (20)	Roche Cobas8000-c702	21.0 µmol/L	99.9%	0.01%	100.0%	185,791	88%
Lippi *et al*. (21)	Roche Cobas8000	21.0 µmol/L	99.7%	0.10%	100.0%	13,024	49%
Torrado Carrión *et al.* (18)	Roche Cobas8000-c701	34.2 µmol/L	95.7%	NA	99.7%	31,161	88%
Salinas *et al.* (16)	Roche Cobas c711	34.2 µmol/L	96.5%	1.20%	99.8%	100,207	94%
Szoke *et al.* (17)	Roche Cobas c501	34.2 µmol/L*	99.6%	0.10%	99.9%	33,657	NA
Szoke *et al.* (17)	Roche Integra 800	34.2 µmol/L^†^	88.7%	2.10%	97.4%	44,474	NA
Pasqualetti *et al.* (19)	Abbott Architect c16000	13.7 µmol/L*	99.6%	0.10%	99.7%	18,486	≈35%
Pasqualetti *et al.* (19)	Abbott Architect c16000	13.7 µmol/L^†^	98.6%	0.20%	99.4%	3700	≈40%
This study	Abbott Alinity c	15.4 µmol/L; 17.1µmol/L	97.0%	0.28%	99.6%	22,485	66%
* Serum. ^†^Plasma. FN – false negative results. NPV - negative predictive value. NA - not available. I-index – icteric index. Tbil – total bilirubin. NA – not available.							

Regarding the second objective of this study, we set I-index cut-off with the highest specificity and lowest false positive results, finding similar results than others studies with I-index ≥ 34.2 µmol/L (*16*-*18*). Hyperbilirubinemia diagnosis could be important in these cases to know the subjacent aetiology, such as Gilbert syndrome, hepatocellular damage, biliary obstruction, haemolytic anaemia, or others (*12*). One of these disorders, Gilbert syndrome, which has a prevalence of 2.4% to 8% in general population could be reflected in these results (*22*, *23*). The analysis of TBil in samples without TBil requested would have a minimal economic repercussion (30.84 € in two months, 0.069 € per test) and we will get a TBil result ≥ 20.5 µmol/L in 99% of cases. Nonetheless, we need to know that if we inform TBil we could be generating an overdiagnosis, which could lead to inappropriate testing (radiological, laboratory, *etc.*) and could result in bigger cost without patient being diagnosed with some specific disease or with disease that do not need any intervention (like Gilbert syndrome). On the other hand, a previous revision of patient´ medical records and performing laboratory liver tests could help us to decrease the overdiagnosis.

An important issue that has not been previously studied is how haemolysis could affect to I-index determination. We have shown that the haemolysis decrease sensitivity and increase false negative rate when we apply rejection cut-offs. For this reason, we recommend to determine TBil independent of I-index value in these cases. On the other hand, haemolysis seems to have not interference to diagnose hyperbilirubinemia.

**Figure 1:**
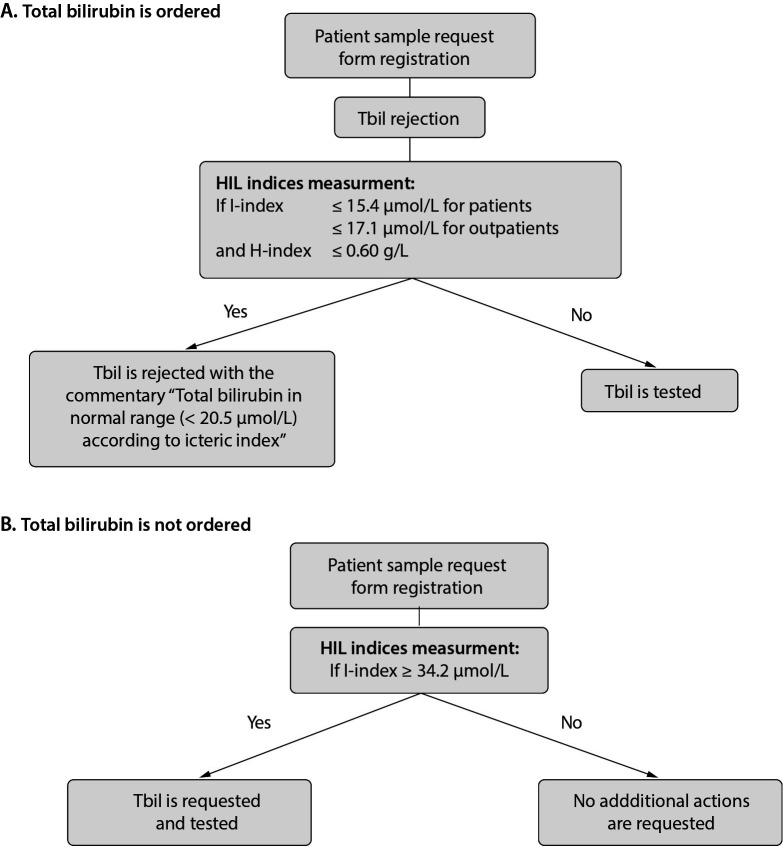
Schematic representation of the rules implemented on LIS to manage total bilirubin according to serum indices. A) Total bilirubin is requested and B) is not requested in patient sample request forms. I-index – icteric index. Tbil – total bilirubin. H-index – haemolysis index. HIL - haemolysis, icterus, lipemia indices

Different rules can be applied on LIS to manage TBil determination (Figure 1). It is important to note that the implementation of these strategies should be cautiously planned and a consensus between laboratory professionals, requesting physicians and health care institutions should be reached.

This study is not intended to replace TBil determination by I-index. One limitation of this study may be related to the fact that the I-index is still not approved by The Food and Drug Administration and insurance companies in some countries may not reimburse a non-performed test or do not assume the addition of other laboratory tests (*16*). The sample size was not as large as other studies but gave an adequate power for area under the curve analysis (*16*, *20*). We extracted data from two months only. It would be more appropriate to work with data from 1-year period since it could minimize summer oscillations, when the workload is usually decreased, especially in primary care. Nowadays, both internal and external quality controls are available for most of the countries. The Alinity version used in this study did not allow to configure internal quality controls for I-index as quality control samples. As long as this is not corrected, these strategies should not be applied in clinical laboratories, although external quality controls are used (Figure 1). The findings presented in this work can be considered preliminary and should encourage other laboratories to validate the accuracy and effectiveness of the proposed approach. Another important issue is the lack of harmonization between manufacturers (*15*). Future multicentre studies should be performed to show that the icteric index is ready to be used in daily practice as a marker to select patients for TBil determination. Finally, we must be aware that the introduction of new rules in the LIS could modify the turnaround times (TAT). For this reason, the next step in implementation of these strategies is the study of the effect in TAT in core laboratories.

In conclusion, this study supports the use of I-index to avoid bilirubin determination and to identify patients with hyperbilirubinemia. This work considers that the economic and test savings could help increasing the efficiency in clinical laboratories.
